# Steady-State Conduction Current Performance for Multilayer Polyimide/SiO_2_ Films

**DOI:** 10.3390/polym13040640

**Published:** 2021-02-21

**Authors:** Muhammad Shoaib Bhutta, Shakeel Akram, Pengfei Meng, Jerome Castellon, Serge Agnel, Hui Li, Yecai Guo, Ghulam Rasool, Shahid Hussain, Muhammad Tariq Nazir

**Affiliations:** 1 Binjiang College, Nanjing University of Information Science & Technology, Wuxi 214105, China; shoaibbhutta@hotmail.com (M.S.B.); hitlihui1112@163.com (H.L.); guo-yecai@163.com (Y.G.); ghulam46@yahoo.com (G.R.); 2 College of Electrical Engineering, Sichuan University, Chengdu 610065, China; shakeel.akram@scu.edu.cn; 3 Institut d’Electronique et des Systèmes, University of Montpellier, 34090 Montpellier, France; jerome.castellon@umontpellier.fr (J.C.); serge.agnel@ies.univ-montp2.fr (S.A.); 4 School of Materials Science and Engineering, Jiangsu University, Zhenjiang 212013, China; shahid@ujs.edu.cn; 5 School of Mechanical and Manufacturing Engineering, University of New South Wales, Sydney, NSW 2052, Australia; tariq.nazir@unsw.edu.au

**Keywords:** polymer nanocomposites, space charge limited current (SCLC), Poole–Frenkel, conduction current, multilayer insulation

## Abstract

The steady-state electrical conduction current for single and multilayer polyimide (PI) nanocomposite films was observed at the low and high electric field for different temperatures. Experimental data were fitted to conduction models to investigate the dominant conduction mechanism in these films. In most films, space charge limited current (SCLC) and Poole–Frenkel current displayed dominant conduction. At a high electric field, the ohmic conduction was replaced by current–voltage dependency. Higher conduction current was observed for nanocomposite films at a lower temperature, but it declined at a higher temperature. PI nanocomposite multilayer films showed a huge reduction in the conduction current at higher electric fields and temperatures. The conclusions derived in this study would provide the empirical basis and early breakdown phenomenon explanation when performing dielectric strength and partial discharge measurements of PI-based nanocomposite insulation systems of electric motors.

## 1. Introduction

Polyimide (PI) films are a widely used insulating material for engineering industries. These thin films are mainly used in electronic devices, multilayer surface coatings on metals, coatings on intermetallic compounds, temperature protection blankets in space crafts and magnetic wire enamelling for electric motor insulation [1,2]. PI thin films are also used for sensor coatings and composite electrodes for batteries [3,4,5,6,7,8]. PI nanocomposite films have also been studied and reported in different well-reputed research articles [9,10,11,12]; however, among these well-documented research articles, most of the focus is on the electrical breakdown strength related to the charge trapping and de-trapping mechanism and mechanical breakdown strength [13,14,15,16,17,18]. Despite this, very few studies are reported in relation to the early breakdown phenomenon in these films.

Over the last decade, nanodielectric insulating materials have received a lot of attention in the global market [19,20,21,22]. Several published studies proclaim that polymers and their derived nanocomposite insulating materials can enhance the dielectric properties for electrical applications [23,24,25,26,27,28,29]. In another study, fluorine-coated PI films have shown promising improvements in results by applying a thin layer of conductive surface coating on the top and bottom of these films [30,31]. These huge improvements in dielectric properties are conditioned on a better dispersion and interface region of nanoparticles, which is not easy to achieve, because these tiny particles can easily agglomerate. Other conditions necessary to obtain better results from these polymer-based nanocomposite materials are the structure of the composite and the size and type of nanoparticles being carefully selected and dispersed. Initially, it seemed as though nanoparticles were highly effective at improving the polymer’s properties; this would later be proven wrong after discerning the correct chemistry and mechanism of polymer-based nanocomposites. However, nanoparticles can enhance the polymer’s properties if important parameters, as mentioned above, are considered. The potential for these advancements has motivated us to improve the dispersion of nanoparticles and dielectric properties by synthesizing the multilayer structure. Therefore, we further explored those mechanisms in this research field using experimental and simulation work.

Conduction Current Theory

The sum of all conduction currents gives us a complete polarization current, which can be given as Equation (1) [32,33,34] where *i_i_* is the instant current, *i_a_* denotes the dipoles’ relaxation current and *i_c_* is the impurities conductivity current.

(1) $$i_{p}=i_{i}+i_{a}+i_{c}$$

We can use Equation (2) to describe the conduction phenomenon. The time needed to obtain the steady-state current relies on the material’s nature, the applied electric field and the temperature. Normally, at higher temperatures and electric fields, the steady state is achieved earlier.

(2) $$\sigma_{t}=\;\sum n_{i}.q_{i}.\mu_{i}$$

where *n_i_* is the concentration of charge carriers, *q_i_* are the fundamental charges and *μ_i_* represents the respective charge carrier’s mobility. Under the electric field, the charge carrier moves either in the direction of the field or the opposite direction, depending on the dominant carrier polarity. For higher temperatures, the charge carriers and the charge mobility increase because the molecular motion increases with temperature. So, the following Arrhenius law from Equation (3) can describe the relationship between charge density, mobility and specific temperature *T*:

(3) $$n\;.\;q\sim \exp(\frac{-E_{A}}{kT})$$

where *E_A_* is the thermal activation energy and *k* is the Boltzmann constant. According to this law, the conductivity increases with the increase in temperature. The field strength can also affect the density and mobility of the carriers, though it is generally believed that it is true only for electrons. The measurement of conductivity as a function of field strength provides a way to distinguish between mechanisms controlled primarily electronically and secondly by ions or dipoles [34,35]. The electrical conductivity increases with the increase in temperature and electric field, but, at the same time, it can be limited by the presence of intrinsic space charges because intrinsic charges can be understood as traps for the moving charges [36,37]. If homocharges are injected at the interface between the dielectric and the metal electrode, the local field is lowered, determining an increase in injection phenomena. Mott and Gurney have proved that the maximum current density *J* that corresponds to the space charge saturation (filled traps) inside the material, in a perfect dielectric without intrinsic carriers and without electron holes, will be given by Equation (4) [38].

(4) $$J=\;\frac{9}{8}\varepsilon \mu \frac{V^{2}}{d^{3}}$$

This equation is valid only if the internal field completely cancels the electric field at the interface between the dielectric and the metal due to space charges. If the interfacial field is present, then the maximum current density *J* will be from Equation (5),

(5) $$J=\;\frac{9}{8}\varepsilon \mu V^{2}{\left[{\left(d+x0\right)}^{3/2}-x_{0}{}^{3/2}\right]}^{-2},$$

where *x*_0_ is the number of free electrons at the anode. For the case of a perfect dielectric, if the external field is low, the current density has an ohmic behavior, as presented in Equation (6).

(6) $$J=\;\sigma \frac{V}{d}=qn_{0}\mu \frac{V}{d}$$

If we compare the previous case with that of perfect insulation, the current density will be reduced with a specific factor *θ*, which corresponds to the fraction of carriers that are injected and trapped in the dielectric, as shown in Equation (7).

(7) $$J=\;\frac{9}{8}\varepsilon \mu \theta \frac{V^{2}}{d^{3}}$$

**Figure 1:**
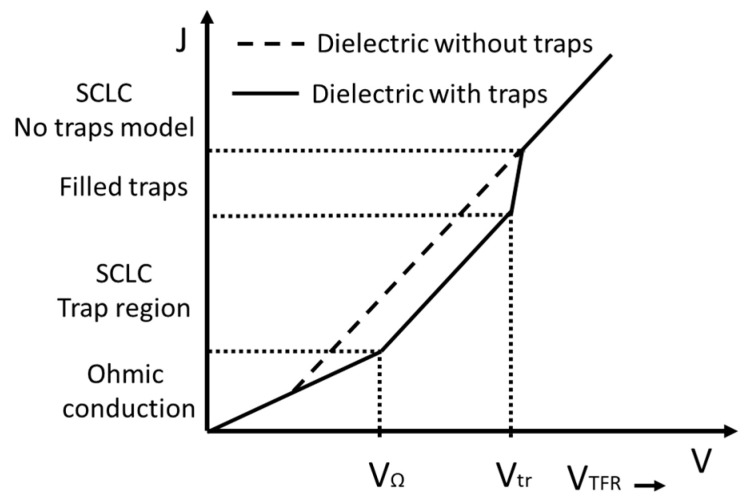
Conduction current density vs. voltage.

In the case of trapped charges, the depth level of the trapped charges also needs to be considered [39,40]. If the sample contains only one level of charge traps, the transition between the ohmic conduction and the space charge limited conduction is possible under the influence of a high field, due to part of the injected charges being trapped. However, when all the charge traps are filled, for a specific electric field (given by the trap filled region applied voltage V_TFR_), a sudden increase in current will be observed because the current will tend to approach the current density of material without traps. The two phenomena may be repeated if the material has several trap levels. The value of the V_TFR_ voltage is given by Equation (8),

(8) $$V_{TFR}=\;\frac{qd^{2}n_{t}}{2\varepsilon },$$

where *n_t_* represents the density of the traps found in the material. The polarization current for the samples increases as the electric field increases because the charge injection and the charge mobility depend on the electric field. The presence of a space charge inside the bulk of the samples can act as traps for the moving charges and limit the charge transport phenomena. Two phenomena can be used to describe the conduction for dielectric materials: the Poole–Frenkel effect and the space charge limited current (SCLC) [37,41]. Suppose that the applied electric field is low and the charge injection is neglected. In that case, as Coelho explains, the variation in the current density (*J*) concerning the electric field (*E*) corresponds to an ohmic behavior [34,42,43]. As the applied electric field increases, the type of conduction changes after a certain threshold voltage. The initial ohmic conduction is changed to space charge conduction, which depends on the trapped charges’ depth. When all the charge traps are filled, a sudden increase in current should be observed for a specific electric field, and the current will tend to reach the current density of material without traps [44]. The schematic diagram of conduction current J vs. E is presented in Figure 1.

## 2. Experimental Section

### 2.1. Sample Synthesis Procedure

The polymer chain can be obtained by reacting two monomers. In our case, we obtained Polyamic Acid (PAA) by reacting two monomers—diamine (ODA) and Pyromellitic dianhydride (PMDA)—in dipolar aprotic solvents, such as N-methyl pyrrolidone (NMP) or N, N-dimethylacetamide (DMAC), which later transformed into the final PI films by applying thermal imidization to PAA solution from 60 °C to 300 °C. The thermal and mechanical properties of PI can be altered by adopting several available monomers. During the reaction process, the percentage of ODA and PMDA can alter PAA’s molecular weight [43,45]. For PAA with a high molecular weight, it is important to dry the absorbed moisture from PMDA at 100 °C. First of all, ODA was put into the beaker and blended with DMAC for 30 min. The electromechanical system was used to stir the solution, then PMDA was blended into the mixture in two portions (first 90%, then the remaining 10%). It was mixed further for 8 to 10 h to obtain the final product of the PAA solution for PI films [45].

**Figure 2:**
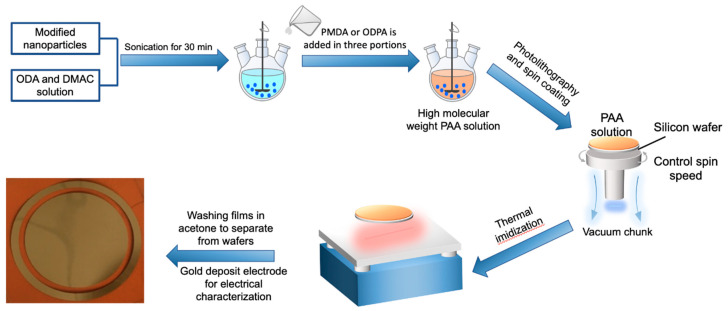
Synthesis process of Polyamic Acid (PAA) solution. Polyimide (PI) films curing process using a spin coating technique and electrode deposition.

The PAA/SiO_2_ nanocomposite solution synthesis process is shown in Figure 2. The surface of SiO_2_ was modified by using the KH-550 coupling agent to produce the chemical linkage between organic PI and inorganic SiO_2_ nanoparticles. After modification, SiO_2_ nanoparticles were dried and blended with DMAC under ultrasonic waves for 30 min, then ODA was added and blended further for 60 min. Then, PMDA was mixed in two parts (90% and 10%) and blended for 6–8 h to get a yellow-colored nanocomposite-based PAA solution.

### 2.2. Spin Coating Technique to Cast PI Films

The spin coating technique was used to stake the PAA solution onto substrates [12]. Typically, a PAA solution is poured on the vacuum chunk substrate and rotated at high speeds of up to 6000 rpm. The control of the spinning speed helps to distribute the PAA solution to the entire surface homogenously. Two to three steps of speed rise can be used to allow the PAA solution to gradually cover more than 80% of the substrate before continuing on to the final speed. For multilayer PI/SiO_2_ films, two spinning speed levels were applied. After calibration, we applied 20 s at a speed of 500 rpm to obtain the first PI layer of 60 µm thickness, and then soft baked it for 30 min at 80 °C. Once the first layer was half cured on the silicon wafer, the nanoparticles-mixed PAA solution was then poured on the half-cured first layer and we placed the silicon wafer on the spin coating machine; a spin speed of 1000 rpm at 30 s was applied to obtain the second PI/SiO_2_ layer of 20–30 µm thickness [45].

### 2.3. PI Films Fluorination

An F_2_/N_2_ gas phase mixture with 12.5% F_2_ by volume at 0.1 MPa pressure and 40 °C temperature was used for 45 min in a laboratory-made stainless steel vessel to modify the surface on both sides of PI films. The thickness of the PI was 125 μm and the thickness of the fluorination coating was 0.5 to 1 μm [30,46,47]. Measured samples have deposition electrodes of 5 cm diameter on both sides.

### 2.4. Composition of Multilayer Films and SEM

**Figure 3:**
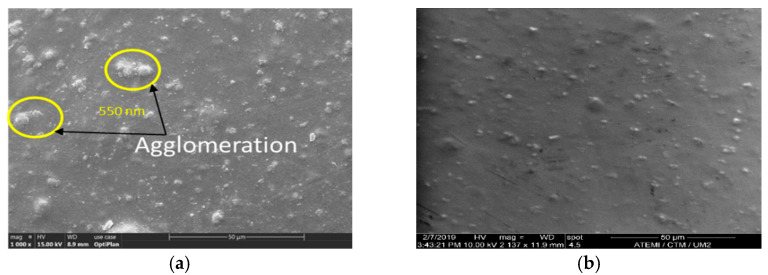

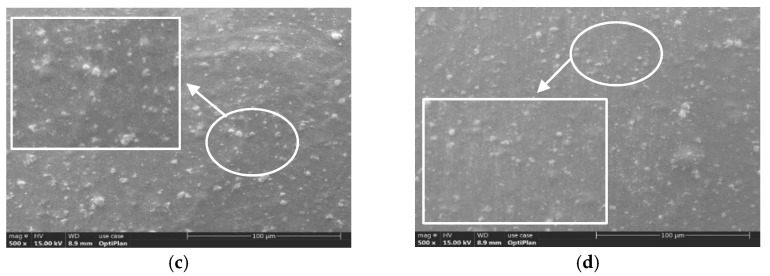
SEM image of surface topography of single PI/SiO_2_ films (**a**,**c**) and multilayer PI/SiO_2_ nanocomposite films (**b**,**d**).

Polyimide nanocomposites are thin nanofilms with complex chemical synthesis processes to achieve samples 100 microns or thicker. JEOL JSM 6460 and FEI inspect S50 SEM(JEOL, Tokyo, Japan) at CTM-IES of UM are used to scan PI and multilayer PI/SiO_2_ samples. Scattered secondary electrons are used in SEM for surface topography and composition of samples. In SEM, the sample is coated with a conductive material or pasted on conductive metals such as gold and aluminum. SEM can also provide surface roughness information of samples. The surface area, ranging from 1 cm to 5 µm, can be imaged in a SEM scanning mode with amplification from 20× to 30,000× and a spatial resolution of 50 to 100 nm. The surface topography of the PI/SiO_2_ single and multilayer films is shown in Figure 3. The SEM images in Figure 3a,c show nanoparticle dispersion for single-layer PI/SiO_2_ films while the right column, as shown in Figure 3b,d shows the multilayer PI/SiO_2_ structure. White regions in these figures correspond to the nanofiller inclusions and darker ones to the polyimide matrix. We observed a better nanoparticles dispersion in PI/SiO_2_ multilayer films, as shown in the right column SEM images of Figure 3b,d, compared with PI/SiO_2_ single-layer film with few nanoparticle agglomeration spots with the size of 550 nm, as shown in Figure 3a.

### 2.5. Measurements

**Figure 4:**
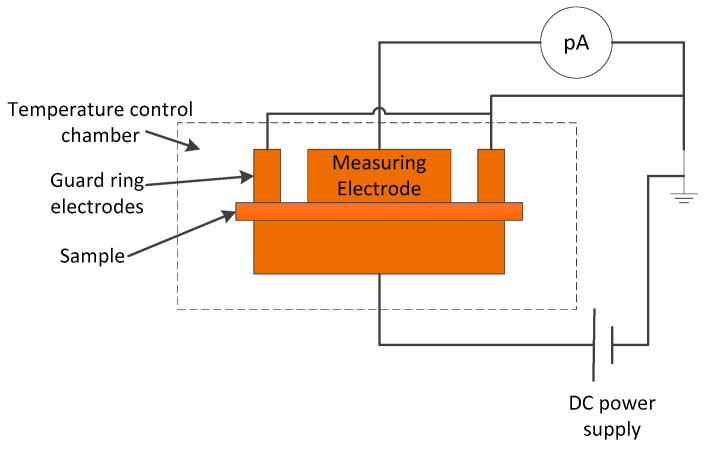
Guard ring electrode setup to measure the conduction current.

**Figure 5:**
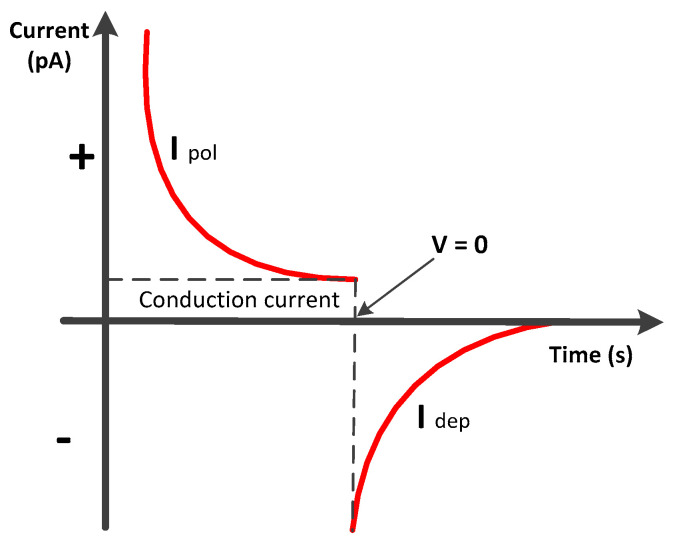
I(t) characteristics of polarization and depolarization current.

The PI films with guard ring electrodes were fixed in a temperature-controlled chamber to measure the conduction current, as shown in Figure 4. The electric field (up to 50 kV/mm) was applied by a low residual ripple voltage power supply with a maximum limit of 35 kV High Voltage DC supply (Fug HCP140-35000). The quasi-steady-state polarization current, known as conduction current, was obtained after the transient regime of the absorption during 3000–5000 s, as shown in Figure 5. When we applied an electric field to the thin sample of PI films, there was slight current conduction through the samples. This current conduction can be due to various reasons [34,44] such as:

(1) the orientation of dipoles;
(2) displacement of the positive and negative charges;
(3) shifting of mobile positive and negative carriers (Maxwell-Wagner-Sillars polarization); and
(4) space charge injection from electrodes and its accumulation in the bulk of the sample.

## 3. Results

**Figure 6:**
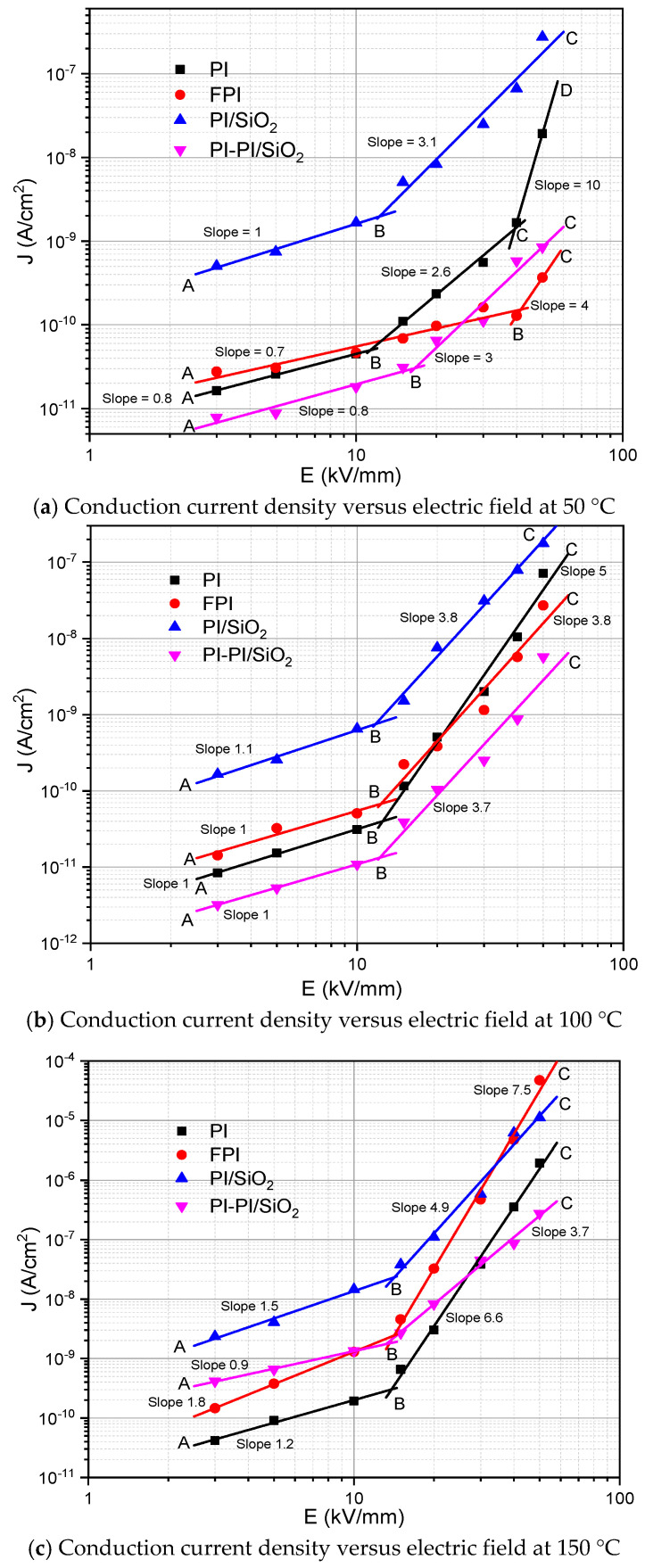
Conduction current density versus electric field at different temperatures.

For the studied conduction current of samples (films), the J vs. E graphs are plotted in Figure 6a–c. By analyzing these, we can observe an increase in conduction current in the case of fillers added in the polyimide matrix at low temperature, but we observe a conduction current decrease for nanocomposite samples at high temperature. All the films expose the same trend of conduction current profile at low electric fields (<16 kV/mm), in which the conductivity of single-layer PI/SiO_2_ and fluorinated samples (FPI) is higher than the pure PI and multilayer PI-PI/SiO_2_ films at low electric fields [47,48,49]. Higher conductivity can help to release trap charges [48,50,51]. As shown in Figure 6, we observed that the films have at least two to three types of conduction from the slope of the current density J, relying on the applied electric field and temperatures. An ohmic current is confirmed by a slope close to 1, corresponding to a linear change in the J vs. E plot. Generally, the ohmic current relates to the participation of the intrinsic charges in conduction. The charge injection is increased from electrodes at the higher electric field, and the ohmic conduction becomes current–voltage dependent.

In this particular case, the dependence between the current density and the electric field confirmed by slopes higher than 1 is found. This behavior could be associated with the different conduction phenomena happening in the samples. It is interesting to observe that the single-layer PI/SiO_2_ samples’ current densities are the highest, whatever the electric field and temperature, while multilayer PI-PI/SiO_2_ samples show, in most of the cases, the lowest current densities—except 150 °C. This could demonstrate that the nanoparticles favor the charge flow when agglomerated and reduce charge flow when dispersing homogenously. After plotting the results in a log/log scale, as shown in Figure 6, we observed an ohmic current in most of the samples with slope ≤ 1 at low field, showing in the region AB. The applied threshold electric field of the order of 9 to 15 kV/mm, depending on the temperature, shows a nonlinearity in the conduction current rise with the slope > 1, indicating a nonohmic conduction mechanism.

The results shown in Figure 6 were further analyzed to find the dominant injection or conduction mechanism in all samples, such as space charge limited current (SCLC) and Poole–Frenkel conduction. The Schottky injection can occur due to electrons’ activation energy at the electrode/dielectric interface caused by lowering the energy barrier at the interface. The Poole–Frenkel emission belongs to the conduction due to the electron’s traps in the bulk of insulation. These trapped electrons can gain some activation energy to be de-trapped and participate in the conduction. Another conduction that can increase the nonlinearity (Slope ≥ 2) in J-E plots is due to the SCLC, which can be influenced by the traps. Therefore, these models can apply to the experimental results to explain the conduction mechanism. The results were analyzed using different representations of the conduction current density J concerning the applied electric field E. We divided the results into three regions (marked as AB, BC and CD) having different slopes, which implies that the J–E relation is of the type J ∝ E^n^, where n is the curve slope. The ohmic regions at 50 °C, 100 °C and 150 °C are shown with the AB region in Figure 6.

**Figure 7:**
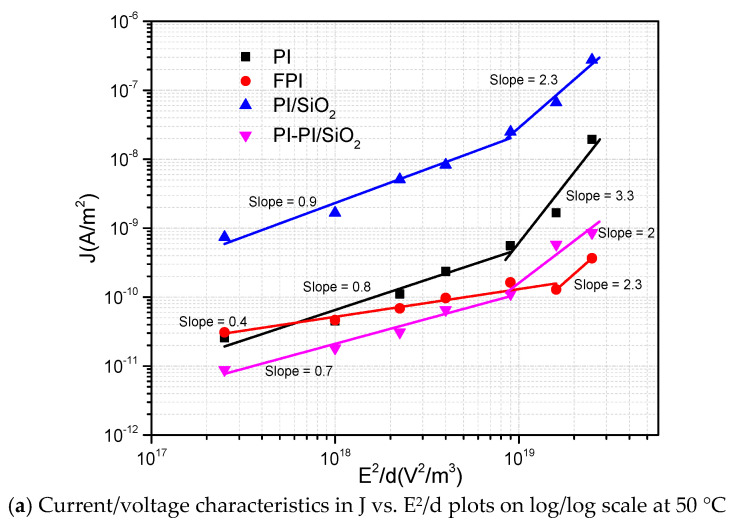

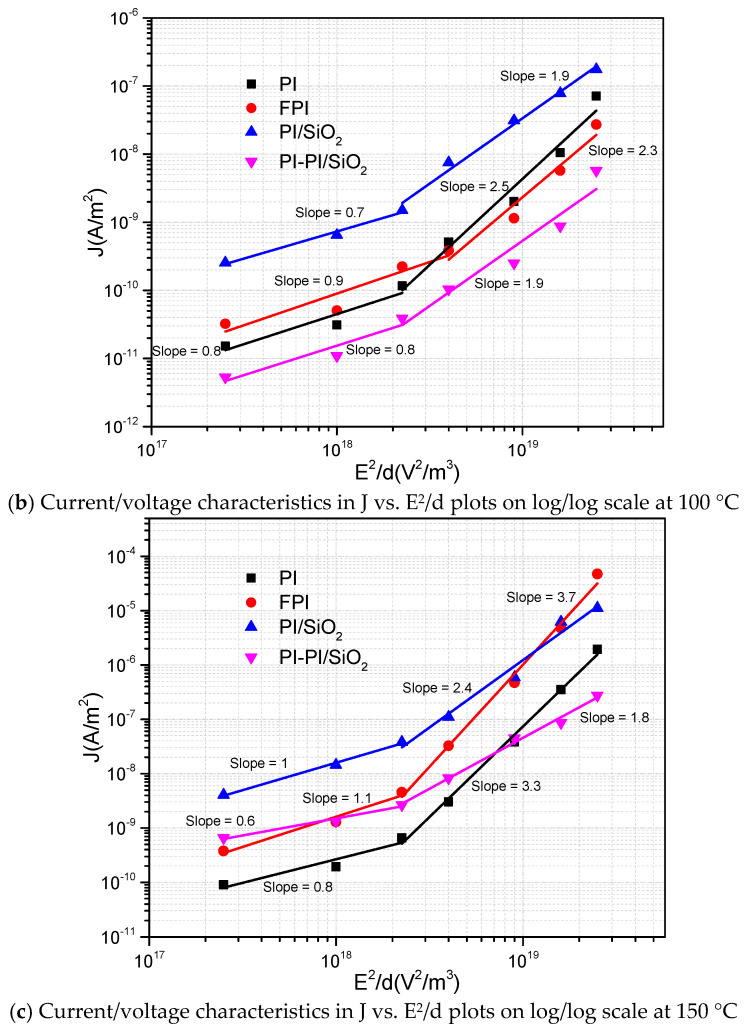
Space charge limited current (SCLC) conduction mechanism at different temperatures.

In Figure 6c, but for FPI and PI/SiO_2_ films at 150 °C, the current density J is proportional to the square of the electric field in the first slope of region AB, which can be the characteristic of an SCLC regime, where the current density–electric field dependence is given by Equation (9) and plotted in Figure 7.

(9) $$J=\left(9/8\right)\varepsilon_{r}\varepsilon_{o}\mu \left(V^{2}/d^{3}\right)$$

where *µ* is the carrier mobility, *V* is the applied voltage, *d* is the sample thickness, *ε_r_* is the relative permittivity of the material and *ε*_0_ is the vacuum permittivity. The slope of the J vs. E^2^/d line should be 1 to confirm the SCLC conduction. Slope <1 in Figure 7 is in area AB, in which the dominant conduction mechanism is ohmic. Thus, it could not be the SCLC mechanism. Slope 1 in Figure 7 represents the trap-free SCLC, whereas slope >1 represents the trap-filled SCLC. Figure 7 shows that only for FPI and PI/SiO_2_ at 150 °C (a better fit for FPI rather than PI/SiO_2_) the slope equals 1, which confirms the SCLC in this region [34]. Thus, it appears that the SCLC mechanism is not the dominant mechanism demonstrated by our samples in the studied conditions.

To conclude, it seems that SCLC would only be present at 150 °C for FPI and PI/SiO_2_ specimens. The second slope for PI, FPI, PI/SiO_2_ and PI-PI/SiO_2_ films obtained at the high electric field has a higher value, which seems to correspond either to the trap-filled region or other conduction mechanisms. The second slope of PI film at 50 °C and the second slope of PI and FPI films at temperatures above 150 °C is higher than 2, which could correspond to other conduction mechanisms. The SCLC regime cannot explain the samples that have a slope region BC higher than 2. The chemical and physical composition of samples related to the glass transition may affect the conduction in these regions.

Thus, the conduction in these regions is mainly controlled by another mechanism. Other conduction mechanisms, such as Schottky and Poole–Frenkel, have to be considered as well. The Poole–Frenkel effect is a bulk conduction mechanism where the barrier between localized states is lowered due to the high electric field’s influence. The conductivity is given by Equation (10) [51].

(10) $$\sigma =\sigma_{o}\exp(\frac{\beta_{PF}\sqrt{E}}{kT})$$

(11) $$\beta_{PF}={\left(q^{3}/\pi \varepsilon_{o}\varepsilon_{r}\right)}^{0.5}$$

where *σ*_0_ is the material’s intrinsic conductivity, *β_PF_* is the Poole–Frenkel constant defined in Equation (11), *k* is the Boltzmann constant and *T* is the absolute temperature. If this mechanism is dominant, the ln (J/E) plot versus E^1/2^ must be a straight line with a slope close to *β_PF_*/(*kT*).

**Figure 8:**
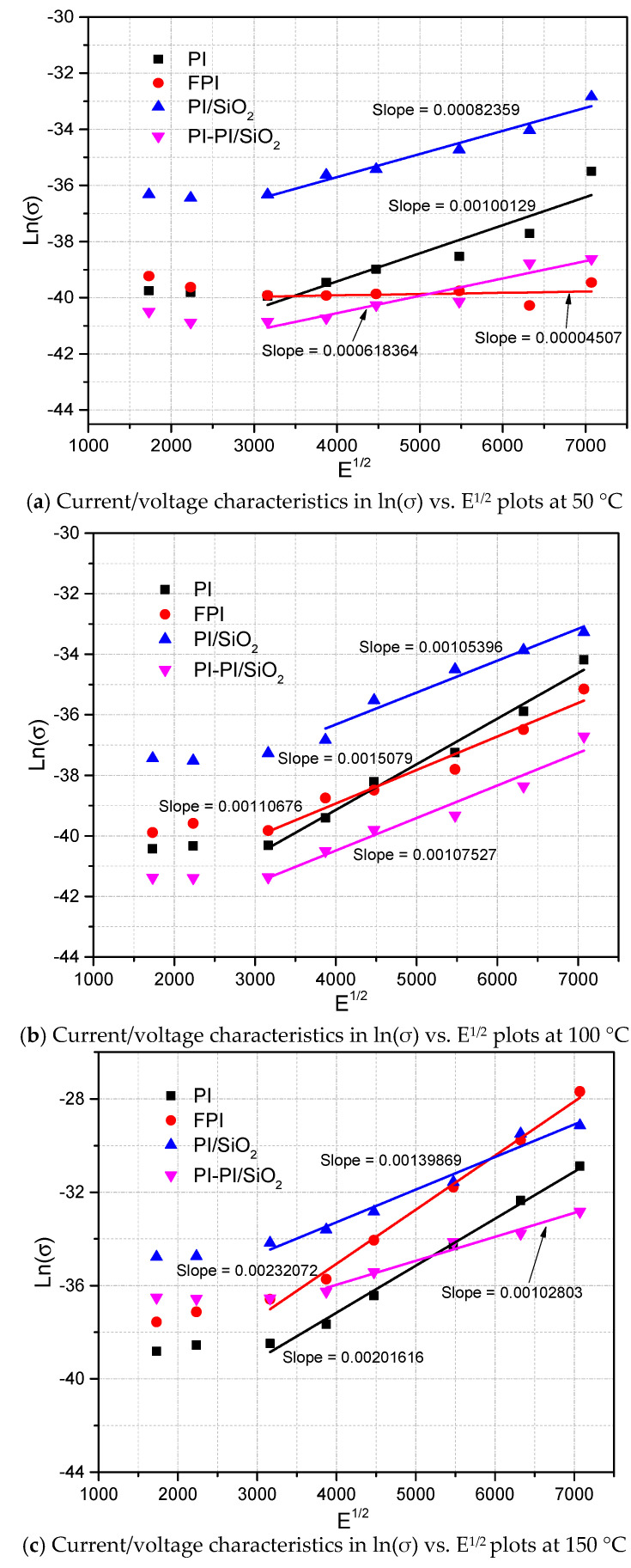
Poole–Frenkel conduction mechanism at different temperatures.

The *β_PF_* coefficients are first calculated from the slope, then the dielectric constants are estimated from these coefficients using Equation (11). If these dielectric constants agree with the values cited in the literature, it could be said that the samples follow the corresponding conduction mechanism. Representations in ln (J/E) versus E^1/2^ coordinates are presented in Figure 8. These representations are not linear for FPI, PI/SiO_2_ and PI-PI/SiO_2_ films at 50 °C, and their dielectric constant is in the range of 6 to 15, which is higher than the measured one.

The slopes of these representations are also not linear for PI, FPI and PI/SiO_2_ films at 150 °C, and their dielectric constant is in the range of 0.8 to 2.2, which is lower than the measured one as shown in Table 1. Therefore, Poole–Frenkel conduction seems not to be the case for these films; however, these representations can be seen to be linear for PI at 50 °C and linear for FPI, PI/SiO_2_ and PI-PI/SiO_2_ at 100 °C, and their values are close to the calculated β_PF_/(kT) values. It is also linear for multilayer PI-PI/SiO_2_ nanocomposite films at 150 °C, and their calculated dielectric constant from the model is close to the measured one, which could confirm Poole–Frenkel conduction in these films.

## 4. Conclusions

Here, we summarize the possible conduction phenomena for all of the studied materials at different temperatures and the electric field ranges. At least one possible conduction of each class related to charge injection or bulk conduction should be present to describe the steady-state conduction current. Nonetheless, for some samples such as FPI and PI-PI/SiO_2_, two different conduction mechanisms from the same bulk conduction class seem to be present at a high temperature of 150 °C. SCLC relates to the mobility of holes and electrons, while other conductions relate to ion donors and acceptor sites present in bulk, which need thermal or electrical energy to participate in the conduction by giving their space to neighboring electrons or holes, depending on their trap energy level. We estimate these conduction phenomena by analyzing the slope of the current density over a certain range of electric field, not just a point. Thus, it seems difficult to consider that this conduction is happening exactly in this electric field. The study of conduction mechanisms in polymeric materials is not easy, and this is even more true when it concerns composite materials. It appears from this study that dominant conduction mechanisms are strongly dependent on the electric field and the measurement temperature, whatever the type of studied material. Nevertheless, in the materials of this study based on polyimides, we can say that two volume conduction mechanisms seem to be predominant at high temperatures.

## Figures and Tables

**Table 1:** Comparison of slope value and calculated β_PF_/kT value at various temperatures.

Samples	ε_r_ (Measured)	Slope	Β_PF_/kT	ε_r_ (Calculated)	Temperature (°C)
PI	3.9	1 × 10^−3^	1.4 × 10^−3^	2.3	50
FPI	3.6	0.4 × 10^−4^	1.4 × 10^−3^	6.1	
PI/SiO_2_	3.95	0.8 × 10^−3^	1.4 × 10^−3^	9.2	
PI-PI/SiO_2_	3.98	0.6 × 10^−3^	1.4 × 10^−3^	15.1	
PI	4	1.5 × 10^−3^	1.2 × 10^−3^	2.4	100
FPI	3.6	1.1 × 10^−3^	1.2 × 10^−3^	4.6	
PI/SiO_2_	3.65	1 × 10^−3^	1.2 × 10^−3^	5.5	
PI-PI/SiO_2_	3.63	1 × 10^−3^	1.2 × 10^−3^	5.5	
PI	3.8	2 × 10^−3^	1.1 × 10^−3^	1	150
FPI	3.6	2.3 × 10^−3^	1.1 × 10^−3^	0.8	
PI/SiO_2_	3.5	1.4 × 10^−3^	1.1 × 10^−3^	2.2	
PI-PI/SiO_2_	3.58	1 × 10^−3^	1.1 × 10^−3^	4.3	

## Data Availability

All data is included within the manuscript.
